# Submucosal Lipoma Causing Small Bowel Intussusception

**DOI:** 10.7759/cureus.17367

**Published:** 2021-08-22

**Authors:** Jordan Roy, Koura Sall, Aphrodite Megaris, Frank DiRoma, Indraneil Mukherjee

**Affiliations:** 1 Surgery, City University of New York (CUNY) School of Medicine, New York, USA; 2 Surgery, Touro College of Osteopathic Medicine, New York, USA; 3 Surgery, Northwell Health, New York, USA

**Keywords:** intussusception, submucosal lipoma, small intestine, small bowel obstruction, lead point

## Abstract

Intussusception involves telescoping of one segment of the intestine into an adjacent segment. Although this diagnosis is common in the pediatric population, it is much less common in adults. One of the main reasons it may occur in adults is due to a mass. Intestinal masses can be malignant, such as gastrointestinal stromal tumors, lymphomas, or adenocarcinomas; or they can be benign. One benign lead point in intussusception is a lipoma. A lipoma usually presents on the trunk, neck, or forearm, but can rarely be seen in the gastrointestinal tract. When it presents in the intestine, it can be either asymptomatic or it can be symptomatic and causes abdominal pain, nausea, vomiting, and gastrointestinal bleeding. Furthermore, it may act as a lead point and causes intussusception. We present an adult patient with two rare findings: small bowel obstruction from intussusception caused by a benign intestinal lipoma as its lead point. The patient was promptly taken to the operating room, where the intussuscepted bowel was resected along with the lipoma, and the patient had an uncomplicated recovery. The pathology report confirmed the specimen to be a submucosal lipoma with mature adipose tissue without atypia. Although intussusception and intestinal lipomas are both rare in adults, it is important to be aware of them on the list of differential diagnoses in adult patients with abdominal pain. This is because it can cause a wide array of complications including, ischemia, bowel perforation, sepsis, shock, and peritonitis. The lead point in intussusception has the possibility of being malignant. Careful consideration of these diagnoses with prompt imaging and appropriate intraoperative management is vital for good patient outcomes.

## Introduction

Intussusception is telescoping or invagination of a part of the intestine into the lumen of an adjacent segment. It is a medical and surgical emergency. It is common in the pediatric population younger than three causing most small bowel obstructions (SBOs) (80-90% incidence) [1]. It can also occur in adults but is a rarer cause of bowel obstruction with an incidence of 1% [2]. There are several types of intussusception, but the most common ones in adults affect the small intestine: jejunojejunal, jejunoileal, ileoileal, ileocolonic, and sometimes colocolonic. In adults, intussusception presents similarly to the pediatric population with sudden-onset abdominal pain that can relax and remit, with or without symptoms of acute small bowel obstruction (SBO) and hematochezia, constipation, or bloating.

Intussusception in adults can be caused by viral infections, polyps, post-surgical adhesions, lipomas, or Meckel’s diverticulum, but there is a greater concern for underlying malignancy as a lead point. This includes gastrointestinal stromal tumors, carcinoid tumors, lipomas, lymphomas, adenocarcinomas, and metastatic disease, each of which can subsequently lead to intussusception. In intussusception, the lead point is pulled forward by normal peristalsis, telescoping or prolapsing the affected segment of the bowel (intussusceptum) into another segment of the bowel (intussuscipiens). Although intussusception is not a common cause of small bowel obstruction in adults, the lead point of small bowel intussusception is of malignant origin in 25% of cases and up to 66% of cases for large bowel intussusception [3-4]. Therefore, it should be considered on a differential and excluded as soon as possible. Even if it is not malignant, intussusception can cause several complications to the bowel sections involved, including bowel ischemia, bowel perforation, sepsis, shock, and peritonitis [2]. Here, we present a rare case of ileoileal intussusception secondary to a submucosal lipoma.

## Case presentation

The patient is a 50-year-old male with a medical history of psoriasis and hypertension and no surgical history who presented to the emergency department at a tertiary care hospital in New York City with a one-day history of worsening diffuse abdominal pain associated with nausea and multiple episodes of non-bloody, non-bilious vomiting. He was afebrile and hemodynamically stable (T: 36.8 ºC, HR: 82, blood pressure, BP: 137/65, RR: 18, O_2_ saturation: 99% on room air) upon arrival to the emergency room. On physical examination, he had diffuse tenderness in all four quadrants of the abdomen, and the nondistended abdomen without rigidity or rebound tenderness. His digital rectal exam revealed an empty rectal vault.

**Figure 1:**
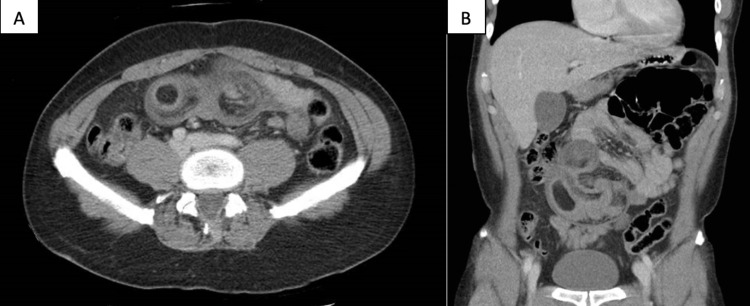
CT imaging. Axial view (A) and coronal view (B) of intussusception within the mid-abdomen with edematous and dilated bowel, compatible with obstruction. The lead point is suspected to be a 3.9 cm small bowel lipoma. No free intraperitoneal air or fluid collection.

His laboratory findings were significant for a white cell count of 11.91 µL and serum lactate of 3.7 mmol/L. The rest of his metabolic panel was unremarkable. A CT abdomen and pelvis with IV contrast revealed a long segment small bowel-small bowel intussusception within the mid-abdomen with markedly edematous and dilated bowel compatible with obstruction. The lead point was suspected to be a 3.9 cm small bowel lipoma (Figure 1A, 1B). 

**Figure 2:**
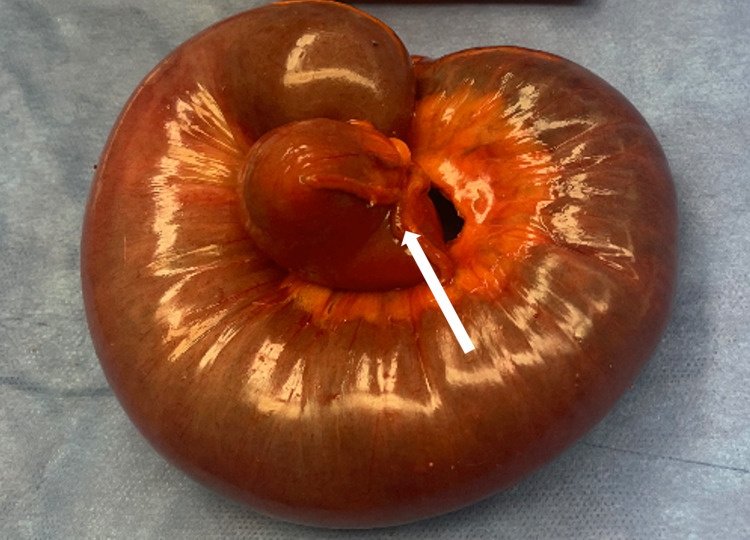
Gross specimen. Gross image shows proximal bowel (intussusceptum) telescoping into distal bowel (intussuscipiens) (white arrow).

A nasogastric tube was placed. He was given hydromorphone and ketorolac. They were unable to control the patient’s abdominal pain. He then underwent an emergency laparotomy where he had a small bowel resection of the intussuscepted bowel loop (Figure 2) and a primary anastomosis of the proximal and distal bowel fragments.

**Figure 3:**
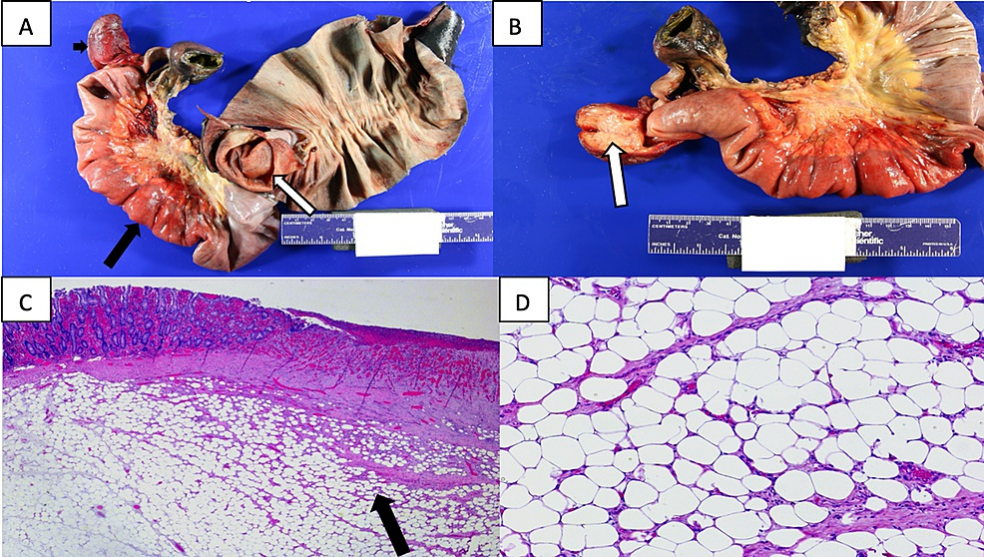
Gross and histopathological features of lipoma causing intussusception. (A) Gross image shows one segment of the bowel (large black arrow) is telescoped into the dilated segment of the bowel (white arrow). Mucosal thickening and multiple areas of erosion (small black arrow) at the lead point of intussusception. (B) Gross image shows the cut surface of mucosal thickening is yellow, soft, and lobulated at the lead point of intussusception (white arrow). (C) On low power, (20 magnification). Segment of the small intestine with ischemic changes, ulceration, congestion, and underlying submucosal lipoma (black arrow). (D) On high power, (400 magnification). Lipoma consists of mature adipose tissue without atypia.

The resected bowel loop measured 13 x 5 cm with attached mesentery measuring 13 x 3 x 2 cm. Its serosal line was pink to gray with a smooth texture. Pathology further examined the specimen and upon opening, one segment of the bowel was telescoped in the dilated segment and measured 24 cm in length (Figure 3A, 3B). The lead point of the intussusception had mucosal thickening and multiple areas of erosion and ulceration and appeared yellow, soft, and lobulated upon opening it. These findings were consistent with ischemic changes, ulceration, and congestion of the small bowel as well as a submucosal lipoma (Figure 3C, 3D).

The patient recovered well and had no post-operative complications. His nasogastric tube was removed on post-operative day two and he tolerated a clear liquid diet, voided, and passed flatus. On post-operative day three, he was advanced to a soft diet, ambulated, and was discharged home. He followed up in the surgery clinic 10 days later with a well healing incision site. He was tolerating his usual diet and having regular bowel movements.

## Discussion

Intraluminal intestinal masses only account for 10-15% of causes of small bowel obstruction after adhesions, hernias, and malignancies, which are responsible for the majority of small bowel obstructions (Table 1) [5]. Of the intraluminal masses, intussusception only accounts for 1% of all cases of small bowel obstruction in adults [2,5]. Thus, it is a rare cause for small bowel obstruction in adults.

**Table 1:** Causes of small bowel obstruction. *Carcinomatosis, endometriosis, inflammatory bowel disease stenosis, ischemic stenosis, gallstones, and foreign bodies [5].

Small bowel obstruction cause	Percentages of cases
Adhesions	55-80%
Hernias	15-30%
Malignancies	5-10%
Others* including intussusceptions	10-15%

In intussusception, the lead point is pulled forward by normal peristalsis, telescoping or prolapsing the affected segment of the bowel (intussusceptum) into another segment of the bowel (intussuscipiens). The lead point of intussusception has several etiologies including, adhesions, polyps, viral infections, lipomas, Meckel’s diverticulum, gastrointestinal stromal tumors, lymphomas, and other malignancies. Although intussusception is not a common cause of small bowel obstruction in adults, the lead point of small bowel intussusception is malignant in origin in 25% of cases and up to 66% of cases of large bowel intussusception [3-4]. Therefore, intussusception should not be ruled out in an adult patient with abdominal pain unless certain. In our case, the lead point of the intussusception causing small bowel obstruction was an intestinal lipoma.

A lipoma is a benign tumor of fat cells that are soft painless nodules that vary in size. They are usually found wherever normal fat cells preside. They arise from mesenchymal cells and often present on the trunk, neck, or forearms [6]. Lipomas can also arise in other less common areas of the body, including the gastrointestinal tract with a reported incidence between 0.15% and 4.4% [6-7]. Intestinal lipomas are more often in the colon (65-75%) and can also be in the small intestine (20-25%) [6]. Intestinal lipomas tend to be asymptomatic and are detected incidentally; however, if they are more than 2 cm, they may become symptomatic and cause GI pathology, such as bleeding, obstruction, or intussusception by acting as a lead point [6]. When lipomas are asymptomatic and are found incidentally, they can many times be left alone. However, if they are large and/or symptomatic, surgical resection offers an excellent prognosis and is the standard method of treatment [6].

When examining an intestinal lipoma, specifically, a small bowel lipoma, there are typically three types: intermuscular lipoma, subserosal lipoma, and submucosal lipoma. Submucosal lipomas are the most common, making up 90% of intestinal lipomas [6]. Submucosal and subserosal lipomas tend to be the ones that cause intussusception, and large subserosal lipomas are prone to causing intestinal compression and volvulus [8]. Furthermore, small bowel lipomas can differ in gross appearance, either being sessile or pedunculated. They can either have regular or lobulated contours [9]. Their overlying mucosa can even have ulceration, especially when in the duodenum--the part of the small bowel exposed to the most gastric acid [9].

The management of intussusception is oftentimes conservative and non-operative, especially in the pediatric population, where it is managed with intravenous fluids and supportive treatment. Barium enemas with air contrast are used to reduce ileocolic intussusception. However, if the intussuscepted intestine has evidence of bowel obstruction, ischemia, or necrosis induced by the lead point, the management is intraoperative intervention to relieve the obstruction and possibly to resect the ischemic bowel as well [10-11]. Likewise, in our patient who had a complete intestinal obstruction with diffuse tenderness, operative management was our first choice.

## Conclusions

The gastrointestinal tract is a relatively uncommon location to find a lipoma, yet when present may either be asymptomatic or symptomatic. When asymptomatic, these lipomas may go unnoticed for years or can be picked up as incidental findings. A large lipoma of the intestine may act as a lead point for intussusception, another rare diagnosis, especially in the setting of small bowel obstruction in adults. Intussusception only represents 1% of all adult SBO cases with less than 4% of cases due to an intestinal lipoma. These patients can present with typical SBO symptoms, such as abdominal pain, nausea, and vomiting.

Our patient had both rare entities presenting as complete small bowel obstruction. It is important to diagnose and treat immediately as these cases can cause necrosis, bowel perforation, and infection, among other potentially serious or lethal complications. The CT scan provided preoperative information that helped to limit the amount of exploration and assisted us in lipoma and small bowel resection and anastomosis, which eventually led to an uneventful recovery. Although uncommon, intussusception must be considered on the differential diagnosis of adult abdominal pain in order to avoid ischemia, and rule out life-threatening gastrointestinal malignancies in adults. Intraoperative management involves reduction of the obstruction and resection of the bowel involving the lipoma and ischemic bowel, when present. Overall, intussusception caused by intestinal lipoma has an excellent prognosis with surgical resection.
